# How to create a scientific journal from scratch – the European Journal of Psychiatric Trainees

**DOI:** 10.1192/j.eurpsy.2023.207

**Published:** 2023-07-19

**Authors:** L. A. Fernandes

**Affiliations:** Psychiatry, Hospital Prof. Doutor Fernando Fonseca EPE, Amadora, Lisbon, Portugal

## Abstract

**Abstract:**

In this workshop we aim to discuss the ins and outs of publishing in psychiatry with practical guidance from both sides of the couch. The executive editors of the European Journal of Psychiatry (Dr Asilay Seker, Dr Mário J. Santos, and Dr Luís Afonso Fernandes) will talk about their experience of starting a journal from scratch with very limited resources and share practical tips with the participants about publishing for early career psychiatrists to early career psychiatrists.

**Disclosure of Interest:**

None Declared

